# Pre- and postoperative offset and femoral neck version measurements and validation using 3D computed tomography in total hip arthroplasty

**DOI:** 10.1177/2058460120964911

**Published:** 2020-10-08

**Authors:** Mats Geijer, Sverrir Kiernan, Martin Sundberg, Gunnar Flivik

**Affiliations:** 1 Department of Radiology, University of Gothenburg, Gothenburg, Sweden; 2 Department of Radiology, Sahlgrenska University Hospital, Gothenburg, Sweden; 3 Department of Clinical Sciences, Lund University, Lund, Sweden; 4 Department of Orthopedics, Clinical Sciences, Lund University and Skåne University Hospital, Lund, Sweden

**Keywords:** Hip arthroplasty, femoral neck anteversion, femoral offset, acetabular offset, proximal femoral symmetry, 3D-measurements

## Abstract

**Background:**

Restoration of a correct biomechanical situation after total hip arthroplasty is important.

**Purpose:**

To evaluate proximal femoral symmetry of acetabular and femoral offset and femoral neck anteversion pre- and postoperatively in hip arthroplasty by semi-automated 3D-CT and to validate the software measurements by inter- and intraobserver agreement calculations.

**Material and Methods:**

In low-dose CT on 71 patients before and after unilateral total hip arthroplasty, two observers used a digital 3D templating software to measure acetabular offset, true and functional femoral offset, and femoral neck anteversion. Observer agreements were calculated using intraclass correlation. Hip measurements were compared in each patient and between pre- and postoperative measurements.

**Results:**

Preoperatively, acetabular offset (2.4 mm), true (2.2 mm), and functional global offset (2.7 mm) were significantly larger on the osteoarthritic side without side-to-side differences for true and functional femoral offset or femoral neck anteversion. Postoperatively, acetabular offset was significantly smaller on the operated side (2.1 mm) with a concomitantly increased true (2.5 mm) and functional femoral offset (1.5 mm), resulting in symmetric true and functional global offsets. There were no differences in postoperative femoral neck anteversion. Inter- and intraobserver agreements were near-perfect, ranging between 0.92 and 0.98 with narrow confidence intervals (0.77–0.98 to 0.94–0.99).

**Conclusion:**

Acetabular and concomitantly global offset are generally increased in hip osteoarthritis. Postoperative acetabular offset was reduced, and femoral offset increased to maintain global offset. 3D measurements were reproducible with near-perfect observer agreements. 3D data sets should be used for pre- and postoperative measurements in hip arthroplasty.

## Introduction

In total hip arthroplasty (THA), restoration of anatomy and seeking for a correct biomechanical situation is crucial for function.^1–4^ Global offset (GO; the sum of acetabular offset (AO) and femoral offset (FO)) and femoral neck anteversion (FNA) are measurements that may be used to evaluate postoperative outcome regarding implant position and degree of restored anatomy in THA. Imbalance in GO may lead to limping. Reduced FO may increase acetabular polyethylene wear.^5^ FNA below 10° appears deleterious to the long-term outcome for cemented stems.^6^

To make as good anatomical restoration and biomechanical situation as possible during THA, it is common to make a preoperative plan on conventional radiographs, so-called templating, to know what implant to use and in what position to insert it. If the affected side, planned for surgery, is too deformed, the planning can be made on the contralateral, hopefully, more normal side. The goal of surgery is to deepen the acetabular socket and reduce the AO to make room for the acetabular component and, at the same time, increase FO to keep GO constant. Asymmetry of the different elements or measurements of the hip joints may, however, render the contralateral hip unsuitable to use for templating, and several articles have reported significant asymmetry of different measurements.^7–9^ Contrarily, a high degree of hip joint symmetry has also been reported.^10^,^11^

**Fig. 1.**
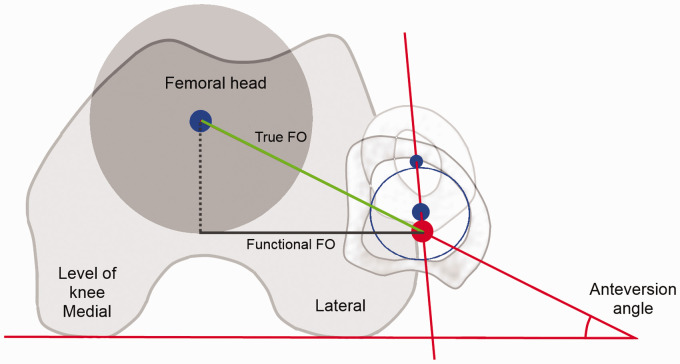
Illustration of the true femoral offset (TFO), which is the true perpendicular distance from the proximal femoral long axis and the rotational center of the femoral head, and the functional femoral offset (FFO), which is the horizontal distance from the proximal femoral long axis and the rotational center of the femoral head. FO: femoral offset.

FO is traditionally measured on anteroposterior (AP) pelvic radiographs,^12^ where several methods of measurement exist.^12^,^13^ It has been shown that measurements on AP pelvic radiographs (here called functional FO) underestimate the true FO^14^,^15^ (Fig. 1) and that it is influenced by hip rotation^16^ and flexion.^17^ The need for more exact measurement techniques has been suggested for more exact templating methods and navigational assistance^18^ at surgery. 3D measurements using CT have provided measurements with high reproducibility.^19^

Measurements of FNA have traditionally been done using conventional radiographs with various methods,^20–22^ which has been seen as sufficiently exact to provide measurements for manual templating and manual orientation of the implants. Calculations in three dimensions (3D)^23^ have provided more precise measurements. Initial CT measurements of FNA differed considerably from measurements done on radiographs,^24^ and different CT measuring methods gave widely different results.^25^,^26^ It has been shown that FNA measurements are dependent on the positioning of the femur for radiographic analysis as well as for two-dimensional CT analysis.^27^ Measurements performed using 3D data sets have shown high consistency for both intra- and interobserver agreements.^28^

The current study aimed to evaluate pre- and postoperative proximal femoral symmetry by semi-automated 3D CT measurements of FNA and the different offsets in a cohort scheduled for THA. A secondary aim was to validate the software measurements by inter- and intraobserver agreement calculations.

## Material and Methods

### Patients

The Regional Ethical Review Board approved the study (2009/369). The current cohort was based on a clinical study, reported elsewhere.^29^ In that study, 75 patients were recruited for THA after informed consent and operated with the uncemented ABG II prosthesis (Stryker Orthopedics, Mahwah, NJ, USA). Due to incomplete imaging for various reasons, there were missing data for two patients preoperatively and two other patients postoperatively. For 71 patients with complete pre- and postoperative CT (45 males, 26 females), mean age was 59.1 years (SD 8.1 years).

### CT examinations

Pre- and postoperative imaging included CT of the hips and knees for 3D assessment of AO, true and functional FO, and FNA. CT was performed on a multidetector helical Brilliance 64 CT scanner (Philips, Eindhoven, The Netherlands). Low-dose settings were used for the preoperative study (hips, CT dose index by volume (CTDIvol) 4.8; knees, 4.2) and slightly higher dose settings for the postoperative study to compensate for the implant (hips, CTDIvol 16.4; knees, unchanged).^30^ Helical CT was performed from the mid-pelvis, including the anterior superior iliac spine to about 6 cm distal to the lesser trochanter, and from directly proximal to the femoral condyles to directly distal to the knee joint. Postoperative imaging covered the same area. Images were archived in the local picture archiving and communication system.

### Image evaluation—3D assessments

**Fig. 2.**
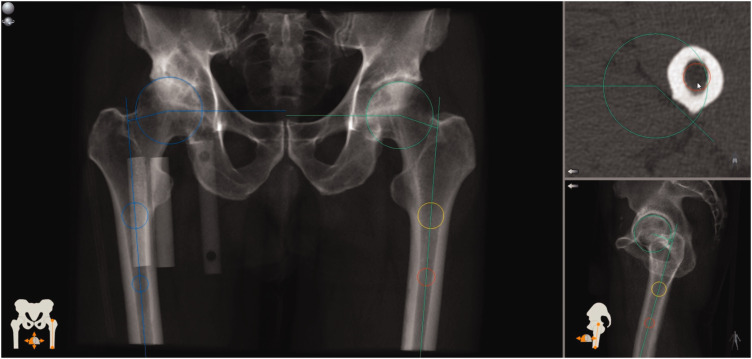
Screen capture showing the best-fit circles defining the femoral head, the definition of the long axis of the proximal femur, acetabular offset, and femoral offset (FO). Functional FO was measured on the coronal reformation, true FO was measured in three dimensions. The external objects at the right hip are bone density phantoms.

The pre- and postoperative 3D-CT examinations were evaluated using a CT-based 3D templating software (Ortoma Plan™, Ortoma AB, Gothenburg, Sweden), giving measurements for AO, true and functional FO, and FNA. The templating software assigned the pelvis and knees CT scan volumes to a combined 3D volume. Thick slab multiplanar reformations (MPRs) were provided in the orthogonal planes by the software (Fig. 2). The pelvis volume was rotated to the anterior pelvic plane^31^ by first defining the bi-ischial line as a horizontal line between the inferior points of the ischial tuberosities or the teardrops on an AP thick slab MPR, secondly the sagittal plane as perpendicular to a line drawn between the anterior superior iliac spines on an axial reconstruction, and thirdly the anterior pelvic plane as a line between the anterior superior iliac spines and the anterior point of the symphysis pubis on a lateral reconstruction. The reconstructed volume was stepwise automatically rotated to the described planes.

**Fig. 3.**
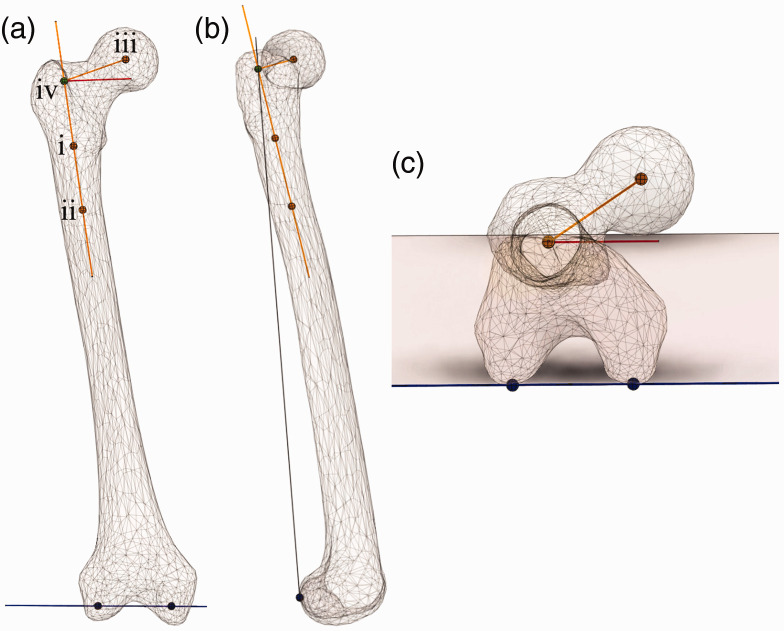
Schematic 3D illustration of the definition of femoral neck anteversion (FNA) in the current study. The long axis of the femur is defined by two points in the proximal femur: (i) at the inferior border of the lesser trochanter and (ii) at a point about 6 cm distal in the femoral shaft. The FNA is the angle between a perpendicular line between the proximal femoral long axis and (iii) the femoral head and a line between the femoral condyles.

The long axis of the proximal femur was defined by assigning best-fit spheres on the AP MPR at the level of the distal border of the lesser trochanter in the CT volume (Figs 2 and 3(a), point i), touching the inner surface of the medial and lateral cortices, and one in the proximal femur about 6 cm distal to the first, proximal to any visible bowing (Fig. 3(a), point ii). The position of the spheres was then adjusted on the lateral and axial views. The rotational center of the femoral head was defined by assigning a best-fit circle on all three thick slab MPRs (Fig. 3(a), point iii). The condylar line of the knee was found by assigning a line tangential to the posterior subchondral joint surface of both femoral condyles (Fig. 3). Both points were then adjusted in the craniocaudal direction on the lateral view to the most dorsal point of the femoral condyles. The condylar plane was defined by the condylar line and the intersection on the long axis for true FO (Fig. 3(a), point iv). A central point in the knee was found at the midpoint of a line drawn between the lateral and medial femoral epicondyle, with the point adjusted in height to the same level as the posterior condylar line. The software then calculated the respective measurements.

AO was defined as the distance from the midline of the symphysis pubis to the rotational center of the femoral head. True FO was defined as the perpendicular distance from the long axis of the proximal femur to the rotational center of the femoral head measured in 3D^12^ (Fig. 1). Functional FO was defined as the perpendicular distance from the long axis of the proximal femur to the rotational center of the femoral head on the coronal MPR, i.e. not taking into account the shortening of the offset due to the FNA, rotation, or flexion of the femur, corresponding to the “classical” FO as measured on conventional radiographs.^32^ FNA was defined as the angle (Fig. 3(c)) between the true FO line and the condylar plane.

Side-to-side comparisons of AO, true and functional FO, and FNA between the non-affected and the osteoarthritic sides were performed on all patients. Postoperatively, the same measurements were repeated to evaluate the surgical outcome.

The measurement technique was validated by performing an interobserver agreement analysis on all pre- and postoperative measurements. For interobserver agreement measurements, two observers (MG and SK) performed the above-mentioned measurements on 71 pairs of hips on all pre- and postoperative studies, 284 measurements per observer.For intraobserver measurements, both observers repeated the measurements on both hips on the pre- and postoperative CT of 15 randomly selected patients after about two months; 60 measurements per observer.

### Statistics

Continuous data are expressed as means and standard deviation. Qualitative data are expressed as frequency and percentage. Observer agreement was analyzed with intraclass correlation (ICC) with 95% confidence intervals (CI). The strength of observer agreement was translated according to definitions proposed by Landis and Koch for kappa values,^33^ as 0.00–0.20, slight; 0.21–0.40, fair; 0.41–0.60, moderate; 0.61–0.80, substantial; and 0.81–1.00, almost perfect. Furthermore, Lee et al.^34^ stated that the lower 95% CI should be above 0.75 for an agreement to exist. The software package R version 3.5.3 was used for statistical computations.^35^

## Results

**Fig. 4.**
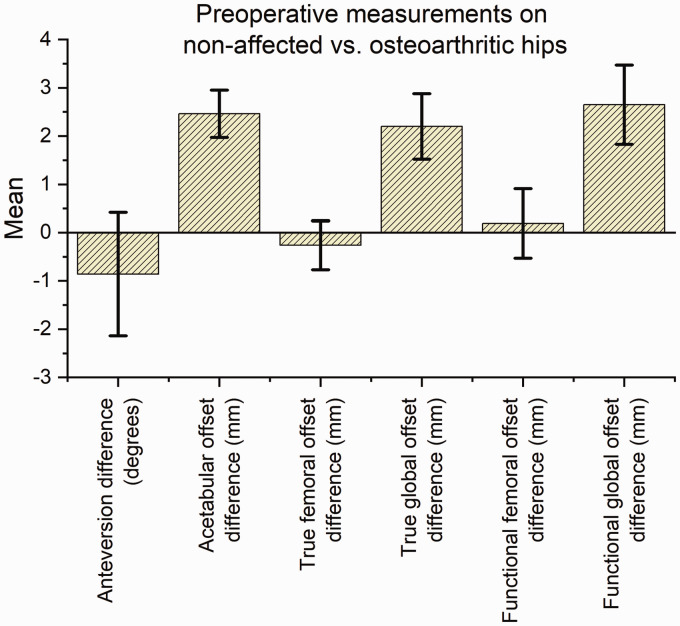
Bar chart showing the differences in measurements between the non-affected side and the side planned for total hip arthroplasty for femoral neck anteversion, acetabular offset, true femoral offset, true global offset, functional femoral offset, and functional global offset. Data for the two observers combined.

Preoperatively, there was a significantly larger AO on the osteoarthritic side of 2.5 mm (95% CI: 1.97–2.95), resulting in correspondingly larger true and functional GO of 2.2 (CI: 1.52–2.88) and 2.6 mm (CI: 1.83–3.47), respectively. There were no significant differences in preoperative true or functional FO or FNA between the non-affected and the osteoarthritic sides (Table 1, Fig. 4).

**Table 1.** Preoperative differences between non-arthritic and osteoarthritic hips in 71 patients. Combined data for two observers.

	Mean	95% CI
Femoral anteversion angle (degrees)	–0.86	–2.14 to 0.42
Acetabular offset (mm)	2.46	1.97 to 2.95
True femoral offset (mm)	–0.26	–0.76 to 0.25
True global offset (mm)	2.20	1.52 to 2.88
Functional femoral offset (mm)	0.19	–0.53 to 0.91
Functional global offset (mm)	2.65	1.83 to 3.47

**Fig. 5.**
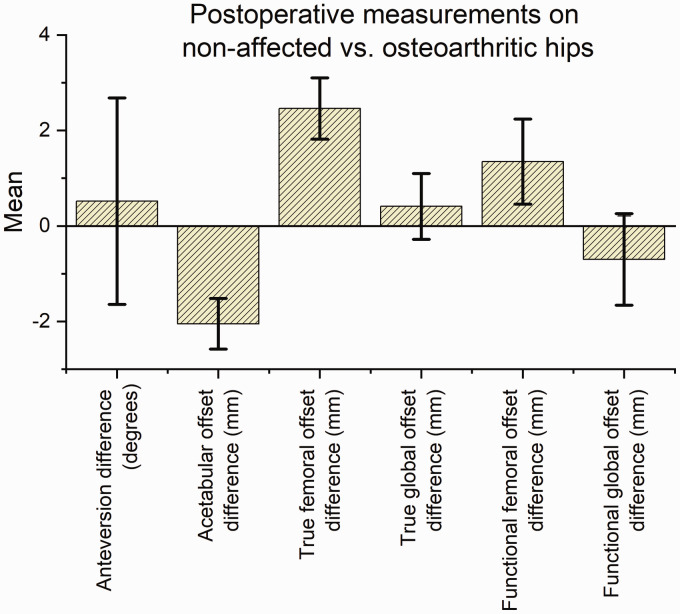
Bar chart showing the differences in measurements between the non-affected side and the postoperative values for the operated side for femoral neck anteversion, acetabular offset, true femoral offset, true global offset, functional femoral offset, and functional global offset. Data for the two observers combined.

Postoperatively, there was a significantly smaller AO on the operated side (–2.0 mm, CI: –2.58 to –1.52) with a concomitantly increased true FO (2.5 mm, CI: 1.82–3.10) and functional FO (1.4 mm, CI: 0.47–2.24) (Table 2, Fig. 5). This resulted in symmetry for both true GO and functional GO, without significant side-to-side differences. There were no significant side-to-side differences in FNA.

**Table 2.** Postoperative differences between non-arthritic and osteoarthritic hips in 71 patients. Combined data for two observers.

	Mean	95% CI
Femoral anteversion angle (degrees)	0.52	–1.65 to 2.68
Acetabular offset (mm)	–2.05	–2.58 to –1.52
True femoral offset (mm)	2.46	1.82 to 3.10
True global offset (mm)	0.41	–0.29 to 1.10
Functional femoral offset (mm)	1.35	0.47 to 2.24
Functional global offset (mm)	–0.70	–1.61 to 0.26

Observer agreements measured by ICC for the two observers were high. Observer agreement for 213 native hips, i.e. 71 pairs of hips on the preoperative CT and the 71 non-operated hips on the postoperative examination, was good with almost perfect ICC scores and narrow CI (Table 3). Observer agreement for the 71 operated hips was equally good with almost perfect ICC scores and narrow CI (Table 3), without differences between pre- and postoperative results. Thus, the results showed no increased difficulty in measuring on postoperative hip examinations. Intraobserver agreements were almost perfect for both observers, with narrow CI (Table 4).

**Table 3.** Interobserver agreement assessed with intraclass correlation (ICC) for measurements of acetabular, femoral anteversion angle, true and functional femoral offset, and true and functional global offset on 213 non-operated hips and 71 operated hips in 71 patients.

Type	Measurement	ICC	95% CI
213 non-operated hips	Acetabular offset	0.94	0.88–0.96
	Femoral anteversion angle	0.93	0.90–0.95
	True femoral offset	0.94	0.92–0.96
	True global offset	0.97	0.96–0.98
	Functional femoral offset	0.96	0.94–0.97
	Functional global offset	0.97	0.97–0.98
71 operated hips	Acetabular offset	0.97	0.96–0.98
	Femoral anteversion angle	0.95	0.93–0.97
	True femoral offset	0.96	0.94–0.98
	True global offset	0.98	0.96–0.98
	Functional femoral offset	0.97	0.95–0.98
	Functional global offset	0.98	0.96–0.98

**Table 4.** Intraobserver agreement assessed by intraclass correlation coefficient (ICC) in pre- and postoperative computed tomo-graphy in 15 patients, i.e. totally 45 non-operated and 15 operated hips.

Type	Measurement	ICC	95 % CI
Observer 1, 45 non-operated hips	Acetabular offset	0.94	0.90–0.97
	Femoral anteversion angle	0.97	0.95–0.98
	True femoral offset	0.97	0.94–0.98
	Functional femoral offset	0.98	0.96–0.99
15 operated hips	Acetabular offset	0.99	0.97–1.00
	Femoral anteversion angle	0.95	0.85–0.98
	True femoral offset	0.93	0.81–0.98
	Functional femoral offset	0.96	0.88–0.98
All 60 hips	Acetabular offset	0.97	0.93–0.98
	Femoral anteversion angle	0.97	0.95–0.98
	True femoral offset	0.96	0.94–0.98
	Functional femoral offset	0.97	0.96–0.98
Observer 2, 45 non-operated hips	Acetabular offset	0.96	0.93–0.98
	Femoral anteversion angle	0.94	0.90–0.97
	True femoral offset	0.93	0.87–0.96
	Functional femoral offset	0.96	0.93–0.98
15 operated hips	Acetabular offset	0.97	0.92–0.99
	Femoral anteversion angle	0.95	0.85–0.98
	True femoral offset	0.92	0.77–0.97
	Functional femoral offset	0.98	0.95–0.99
All 60 hips	Acetabular offset	0.97	0.94–0.98
	Femoral anteversion angle	0.95	0.91–0.97
	True femoral offset	0.93	0.87–0.96
	Functional femoral offset	0.96	0.94–0.98

**Fig. 6.**
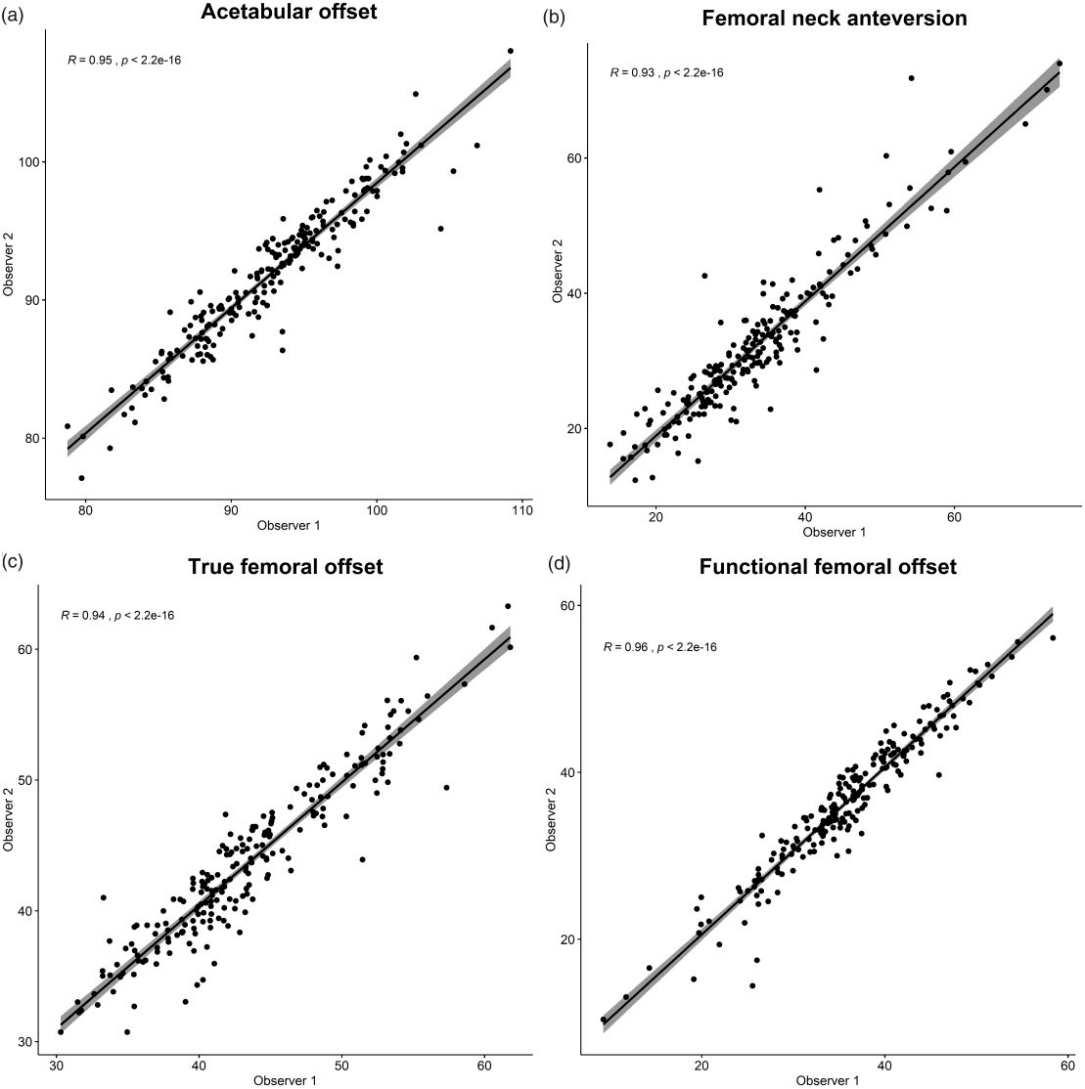
Correlation plots for measurements of acetabular offset, femoral neck anteversion, true and functional femoral offset for two observers in 213 non-operated hips.

Measurements for pre- and postoperative CT of the non-operated hips were compared to evaluate the robustness of the method, i.e. to determine whether repeated CT examinations and measurements on the same hip would yield comparable results. The ICC scores were almost perfect for both observers, with narrow CI (Table 5), and linear regression analyses showed a significant correlation for AO, true and functional FO, and FNA (*p* < 0.001; Fig. 6).

**Table 5.** Comparison of pre- and postoperative assessments in 71 non-operated hips by two observers by intraclass correlation coefficient (ICC).

Type	Measurement	ICC	95% CI
Observer 1, 71 non-operated hips pre- vs. postoperative examination	Acetabular offset	0.94	0.91–0.96
	Femoral anteversion angle	0.93	0.84–0.96
	True femoral offset	0.96	0.93–0.97
	True global offset	0.99	0.98–0.99
	Functional femoral offset	0.97	0.95–0.98
	Functional global offset	0.98	0.97–0.99
Observer 2, 71 non-operated hips pre- vs. postoperative examination	Acetabular offset	0.94	0.91–0.96
	Femoral anteversion angle	0.92	0.88–0.95
	True femoral offset	0.93	0.89–0.96
	True global offset	0.97	0.96–0.98
	Functional femoral offset	0.94	0.90–0.97
	Functional global offset	0.97	0.94–0.98

## Discussion

In the current study, measurements of AO, true GO, and functional GO showed expected results with significant side-to-side differences between the non-affected and the osteoarthritic hips. Postoperatively, AO had been reduced with normalization of true GO and functional GO. The inter- and intraobserver agreements in measuring AO, true FO, functional FO, and FNA with a new hip arthroplasty templating software were generally near-perfect with narrow CI. Measurements on repeated CT examinations were close to identical.

Offset measurements have traditionally been done on AP pelvis or hip radiographs,^12^ where femoral rotation^16^ and flexion^17^ have been shown to influence measurements. In the current study, also true FO was measured, which is the perpendicular distance from the long axis of the femur to the center of the femoral head, measured in 3D. This measurement is always longer than the AP measurement of functional FO and nonaffected by patient positioning.

Measurements of FNA differ between studies, depending on how the measurements were made. Distally, four different methods may be used to define the femoral condylar axis; a line drawn between the most posterior points of the condyles, a line drawn between the most medial and lateral points on the condyles, a line drawn between the center of the centroids of the condyles in cross-section,^36^ or a line bisecting a line through the most posterior points of the femoral condyles and a line through the most anterior points of the distal femur in the same section.^37^ In the current study, the most posterior points of the femoral condyles were used to define the condylar line.

The method for measurements of FNA used in the current analysis is not identical to the definition by Billing.^38^ Billing defined anteversion based on the long axis of the femur as defined by a point centrally in the distal femur. In the current study, anteversion was measured relative to the long axis of the femur proximally, to better correspond to the true insertion site and final position of a femoral prosthetic stem, and not having to compensate for the physiological bowing of the femoral shaft at templating. The proximal point for defining the long axis of the femur has also been defined in different ways. Billing, despite a detailed description of the geometry of the femur and the definition of FNA,^38^ did not define this point. Murphy et al.^25^ defined it as “a centroid of a cross section of the femur at the base of the femoral neck” on an axial CT section “3 per cent” distal to the middle of the lesser trochanter. In the current study, the condylar plane was defined by the condylar line and the intersection of the proximal long axis line and the line for the true FO measurement.

When using conventional CT images, measurements are improved if the sections are corrected for hip and knee flexion as well as adduction of the hip.^39^ This has up until recently been problematic and one reason why previous comparisons of radiography and CT measurements of FNA have not shown superiority for CT.^27^

**Fig. 7.**
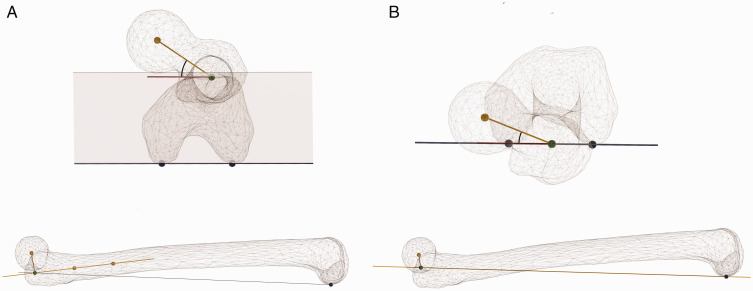
Femoral neck anteversion (FNA) is different depending on the choice of long axis of the femur. (a) In the current study, the proximal part of the femur was chosen for the long axis definition since that corresponds to the length of the femoral stem in hip arthroplasty. FNA is 35°. (b) In previous definitions, e.g. by Billing^38^ and Murphy et al.,^25^ the long axis was defined by a point in the knees and a point in the proximal femur, giving a different measurement value of FNA, 20°.

Since previous studies have used slightly different techniques and measuring points for FNA, the comparison of measurements between studies is difficult (Fig. 7). The most crucial issue, however, is to develop a measuring method with high reproducibility and low observer variation such as in the current study. The high observer agreement rates are consistent with other studies reporting on CT assessment of measurements using 3D images. In one study, reporting on FNA in children using CT and MRI,^28^ ICC values were also near-perfect with narrow CI. In another study measuring FNA directly on 3D reconstructions,^40^ ICC values were high but slightly lower than in the current study. In the current study, CT was performed using a low-dose technique, with an effective dose close to that of radiography,^30^ showing that even with increased image noise, excellent results can be achieved. The results from the current study further support the use of 3D data sets. With the use of 3D data sets, the need for exact patient positioning is practically eliminated.

Various levels of symmetry in normal (non-arthritic) populations have previously been reported regarding femoral hip orientation measurements, seemingly dependent on the evaluated populations. Previously, an asymmetry > 2% has been reported for the femoral neck-shaft angle, FO, FNA, femoral length, and femoral head radius^8^ with substantial differences reported for FNA, FO, and femoral head center location,^7^ all being important measurements for contralateral templating of THA. On the other hand, another study, using high-resolution photographs on cadaveric specimens, reported no significant side-to-side differences for proximal femoral measurements of the femoral head diameter, the minimal neck diameter, and the femoral shaft diameter.^10^

In osteoarthritic hips, reactive bone formation in the acetabular socket often leads to an increase in AO but does not affect FO or FNA. The surgical aim in the current study was to decrease AO with a compensatory increase in FO to restore symmetry in GO. Increasing FO has been suggested to improve abductor strength, reduce limping, and counteract polyethylene wear.^41^ In the current study GO symmetry was achieved according to this principle.

The limitation of the study is mainly the small number of observers. However, the high ICC and narrow CI showed high inter- and intraobserver agreements. There was no reference standard for the measurements, but due to the use of different reference points for measurements in the literature, this was impossible to find.

In conclusion, using low-dose CT with 3D measurements with a templating software yielded excellent repeatability of measurements with near-perfect observer agreement. The study supports the use of 3D data sets for measurements in the pre- and postoperative evaluation in THA.
